# Long-term Shedding of Influenza A Virus in Stool of Immunocompromised Child

**DOI:** 10.3201/eid1607.091248

**Published:** 2010-07

**Authors:** Benjamin A. Pinsky, Samantha Mix, Judy Rowe, Sheryl Ikemoto, Ellen J. Baron

**Affiliations:** Author affiliations: Stanford University, Stanford, California, USA (B. Pinsky, J. Rowe, S. Ikemoto, E.J. Baron);; Willamette University, Salem, Oregon, USA (S. Mix)

**Keywords:** influenza A, infection, pandemics, immunocompromised patient, dispatch

## Abstract

In immunocompromised patients, influenza infection may progress to prolonged viral shedding from the respiratory tract despite antiviral therapy. We describe chronic influenza A virus infection in an immunocompromised child who had prolonged shedding of culturable influenza virus in stool.

Human influenza A virus infections are typically localized to the respiratory tract, and viral presence in the gastrointestinal (GI) tract is rarely observed. Isolation of influenza virus from the stool is most commonly documented in human infections of avian influenza subtype H5N1. In these infections, virus in the stool may be related to a disseminated infection with exceptionally high viral titers, atypical of seasonal, circulating influenza infections (*1*). Interestingly, infections with the influenza A pandemic (H1N1) 2009 virus can be associated with a high rate of GI symptoms (*2*). Although seasonal influenza RNA in stool has been described, we report here the culturing of H1 virus from stool.

## Case Report

The patient was a 4.5-year-old boy who had received a bone marrow transplant for Wiskott-Aldrich syndrome 4 years earlier; he also had chronic graft versus host disease of the GI tract. In January 2008, fever and respiratory symptoms developed in the patient, and direct fluorescent antibody (DFA) testing of a nasopharyngeal swab specimen showed influenza A infection. Over the next several months, he had additional influenza A–positive DFAs and was given oral and aerosolized ribavirin for chronic infection. Despite this aggressive antiviral treatment, >24 respiratory specimens were positive for influenza A by DFA or culture over the next year.

When the patient was ≈3 months into an extended hospitalization at the Lucile Packard Children’s Hospital (complicated by pseudomonal and enterococcal bacteremia, as well as disseminated aspergillosis), he experienced multiple daily episodes of nonbilious, nonbloody emesis (≈2–3/day) and loose stools without blood or mucous (up to 12/day). The clinical team suspected a graft versus host flare but also conducted a work up of the patient to identify an infectious process. Results of blood cultures, stool culture, *Clostridium difficile*–toxin B cytotoxicity assay, and stool examination for ova and parasites were all negative. Results of a urine culture were positive for enterococcus. Culture of stool samples was enterovirus positive and over the subsequent 2 weeks, enterovirus was isolated from 2 additional viral stool cultures. These cultures were not evaluated for the presence of influenza virus. Three weeks after the initial stool testing, results of viral stool culture and repeat stool studies were negative. However, the diarrhea and emesis persisted, and ≈8 weeks after the onset of symptoms, another stool specimen was sent for viral culture.

After 3 days, the culture demonstrated cytopathic effect on primary RhMK cells but not on human foreskin fibroblasts, MRC-5 fibroblasts, or A549 lung carcinoma cells, which is consistent with enterovirus infection. However, results of immunofluorescent staining of the RhMK cells (by using a panenterovirus blend of monoclonal antibodies) were negative. Staining results were also negative with serotype group–specific reagents, including the coxsackie virus B blend, echovirus blend, enterovirus 70 and 71, poliovirus blend, coxsackie virus A9, and coxsackie virus A24 (all enterovirus reagents from Millipore/Light Diagnostics, Billerica, MA, USA). An astute technologist associated the pattern of cells showing a cytopathic effect with the patient’s concurrent influenza A–positive respiratory specimen and long history of influenza infection. She then set up a standard respiratory virus DFA panel (Millipore/Light Diagnostics), which included fluorescein-conjugated antibodies for the detection of influenza A and B; respiratory syncytial virus; parainfluenza 1, 2, and 3; and adenovirus. Strikingly, the specimen was strongly positive for influenza A virus and showed obvious hemadsorption with guinea pig erythrocytes. Two months later, influenza A was again isolated from the patient’s stool, which suggested persistent infection of the GI tract with influenza A virus. Subsequent nucleic acid testing revealed that this chronic influenza A infection was caused by the seasonal, circulating subtype H1N1 virus. Overall, the patient shed influenza A from respiratory secretions for >1.5 years and from stool for >2 months.

## Conclusions

Because viral stool cultures from patients with respiratory infections are infrequently ordered, the true occurrence of influenza virus in stool is unknown. The few studies to date have considered viral RNA in fecal specimens as a marker of GI influenza infection, which may not accurately reflect the shedding of intact virus or the capacity for transmission. One study of 4 children with respiratory symptoms and confirmed influenza infection showed that half had influenza RNA in stool (*3*). In contrast, 6 (<1%) of 627 patients with GI symptoms had detectable influenza RNA in fecal samples (*4*). Similarly, influenza RNA was detected in 21 (2.9%) stool samples from 733 children in Indonesia who had concurrent diarrhea and influenzalike illness (*5*). Notably, in this study, culturable influenza B virus was isolated from the stool of 1 patient. Future studies will be required to ascertain the incidence of influenza virus in the stool of children and adults with influenzalike illness and respiratory influenza infection. Furthermore, given the importance of this issue for infection control and the limited number of laboratories that perform stool viral culture, additional work will be necessary to correlate influenza RNA in feces with the presence of infectious virus.

The lack of a fully intact immune system likely predisposed our patient to chronic influenza infection and spread of the virus to the GI tract. This patient had received a bone marrow transplant for primary immunodeficiency as well as immunosuppressive therapy for chronic graft versus host disease with methylprednisolone, tacrolimus, sirolimus, and daclizumab. Prolonged viral shedding from the respiratory tract and the development of antiviral resistance is well documented in immunocompromised patients, including patients who have received bone marrow transplants (*6*). Although bone marrow transplant patients appear more susceptible to lower respiratory tract disease, in particular, during influenza outbreaks (*7*), influenza virus in stool samples from this patient population has not been well studied. Notably, our patient was not treated with either of the common classes of anti-influenza drugs, the neuraminidase inhibitors or adamantanes, but rather received a long-term course of ribavirin. Although clinical cases of ribavirin-resistant influenza virus infection have not yet been reported, genotypic and phenotypic analysis of this patient’s isolate may show resistance or other virus-specific factors associated with chronic influenza and the presence of virus in stool. Although culturable influenza A virus was isolated from the stool of our patient, whether it played a causative role in the patient’s gastroenteritis could not be determined.

While influenza virus likely spread to the patient’s GI tract after a primary respiratory infection, the route of dissemination remains unknown. One possibility is direct GI inoculation by swallowing respiratory secretions. Because influenza viruses enter the cell through acid-activated fusion with the endosomal membrane (*8*), a low pH environment, for example in the human stomach, is thought to render most influenza viruses noninfectious by prematurely inducing an irreversible conformational change in the viral hemagglutinin (*9*). However, the sensitivity of influenza virus to low pH inactivation appears dependent on strain and subtype (*10*). Our patient was on a proton pump inhibitor, which would reduce gastric acidity. Another possibility is that the virus reached the GI tract hematogenously, as is suspected in human cases of avian influenza (*1*).

Whether the influenza subtype that infected this patient is capable of local GI replication in humans is also unclear. In the GI tract, the virus likely encountered the proteases necessary for hemagglutinin cleavage and activation (*11*). However, the H1 hemagglutinin has relative specificity for α 2,6-linked sialic acid, a cell-surface glyco-conjugate not normally found on mucosa of the colon or small intestine (*12*,*13*). Nevertheless, this binding specificity is not absolute (*14*), and 2,3-linked sialic acids are abundantly expressed on colorectal epithelial cells (*13*). Future studies should assess the ability of influenza viruses to replicate in the human intestinal epithelium.

Early epidemiologic study of the pandemic (H1N1) 2009 virus suggested that it produced diarrhea, vomiting, or both, in ≈25% of case-patients, more often than the previous seasonal, circulating influenza viruses (*2*). Consistent with the GI symptoms of human infection, experimental respiratory inoculation of ferrets with human isolates of the pandemic strain results in high influenza virus titers in the intestinal tract of infected animals (*15*). Because knowledge of transmission of this novel virus is limited, the Centers for Disease Control and Prevention recommends that all bodily fluids, including the diarrheal stool of infected persons, be assumed to be infectious and handled with precautions. With the emergence of this pandemic (H1N1) 2009 strain known to produce GI symptoms, further research addressing the presence of influenza virus in stool could have major consequences for both infection control and disease management.
